# CROPro: a tool for automated cropping of prostate magnetic resonance images

**DOI:** 10.1117/1.JMI.10.2.024004

**Published:** 2023-03-07

**Authors:** Alexandros Patsanis, Mohammed R. S. Sunoqrot, Tone F. Bathen, Mattijs Elschot

**Affiliations:** a Norwegian University of Science and Technology, Department of Circulation and Medical Imaging, Faculty of Medicine and Health Sciences, Trondheim, Norway; b St. Olavs Hospital, Trondheim University Hospital, Department of Radiology and Nuclear Medicine, Trondheim, Norway

**Keywords:** deep learning, image cropping, image processing, prostate cancer, magnetic resonance imaging

## Abstract

**Purpose:**

To bypass manual data preprocessing and optimize deep learning performance, we developed and evaluated CROPro, a tool to standardize automated cropping of prostate magnetic resonance (MR) images.

**Approach:**

CROPro enables automatic cropping of MR images regardless of patient health status, image size, prostate volume, or pixel spacing. CROPro can crop foreground pixels from a region of interest (e.g., prostate) with different image sizes, pixel spacing, and sampling strategies. Performance was evaluated in the context of clinically significant prostate cancer (csPCa) classification. Transfer learning was used to train five convolutional neural network (CNN) and five vision transformer (ViT) models using different combinations of cropped image sizes (64×64, 128×128, and 256×256 pixels^2^), pixel spacing (0.2×0.2, 0.3×0.3, 0.4×0.4, and $0.5\times 0.5\;{\mathrm{mm}}^{2}$), and sampling strategies (center, random, and stride cropping) over the prostate. T2-weighted MR images (N=1475) from the online available PI-CAI challenge were used to train (N=1033), validate (N=221), and test (N=221) all models.

**Results:**

Among CNNs, SqueezeNet with stride cropping (image size: 128×128, pixel spacing: $0.2\times 0.2\;{\mathrm{mm}}^{2}$) achieved the best classification performance (0.678±0.006). Among ViTs, ViT-H/14 with random cropping (image size: 64×64 and pixel spacing: $0.5\times 0.5\;{\mathrm{mm}}^{2}$) achieved the best performance (0.756±0.009). Model performance depended on the cropped area, with optimal size generally larger with center cropping ($∼40\;{\mathrm{cm}}^{2}$) than random/stride cropping ($∼10\;{\mathrm{cm}}^{2}$).

**Conclusion:**

We found that csPCa classification performance of CNNs and ViTs depends on the cropping settings. We demonstrated that CROPro is well suited to optimize these settings in a standardized manner, which could improve the overall performance of deep learning models.

## 1 Introduction

Prostate cancer (PCa) is the fifth cause of death in men and the second most common cancer worldwide.^1^ The current diagnostic procedure for PCa is associated with overdiagnosis leading to overtreatment and misdiagnosis of PCa.^2^ Magnetic resonance imaging (MRI) is used to assist the biopsy procedure when PCa is suspected.^3^ Multiparametric MRI (mpMRI) can improve the detection rate of clinically significant PCa (csPCA) and reduce the overdiagnosis of insignificant PCa.^2^^,^^4^ In addition, the combination of mpMRI with computer-aided diagnosis (CAD) systems can contribute to improving decision-making.^5^ Recently, deep learning has gained significant attention for performing computer vision tasks, such as segmentation, classification, and object recognition.^6^^–^^8^ Convolutional neural networks (CNNs) have shown high performance in medical imaging tasks, such as classification of csPCa,^9^ breast cancer,^10^ lung nodules,^11^ and brain tumors.^12^ Vision transformers^13^ (ViTs) have been shown to outperform conventional CNN models in image classification,^14^ semantic segmentation,^15^ and 3D object recognition.^16^ More recently, ViTs have also shown competitive performance for medical imaging tasks.^17^^–^^19^ Training deep learning models requires a large, annotated dataset. Recent work^20^ has shown that preprocessing of MRI images, such as denoising, MR bias field correction, co-registration, and standardization, improves the performance of classification and segmentation models. However, a subject that has received less attention is how the cropping of these preprocessed images impacts network performance.

Medical images vary in size depending on modality and application, but typically consist of thousands of pixels per slice. Although most deep learning models are adaptable, they have been developed and tested for images of certain sizes, e.g., 32×32,^21^ 64×46,^22^ 128×128,^23^ 256×256,^24^^,^^25^ or images of 512×512^26^ pixels. This forces users to adjust the input to different models.^27^ At the same time, the balance between foreground pixels, representing the region of interest (ROI), and background pixels, representing the region around the ROI, is paramount for deep learning models to achieve robust and accurate results.^28^

**Fig. 1:**
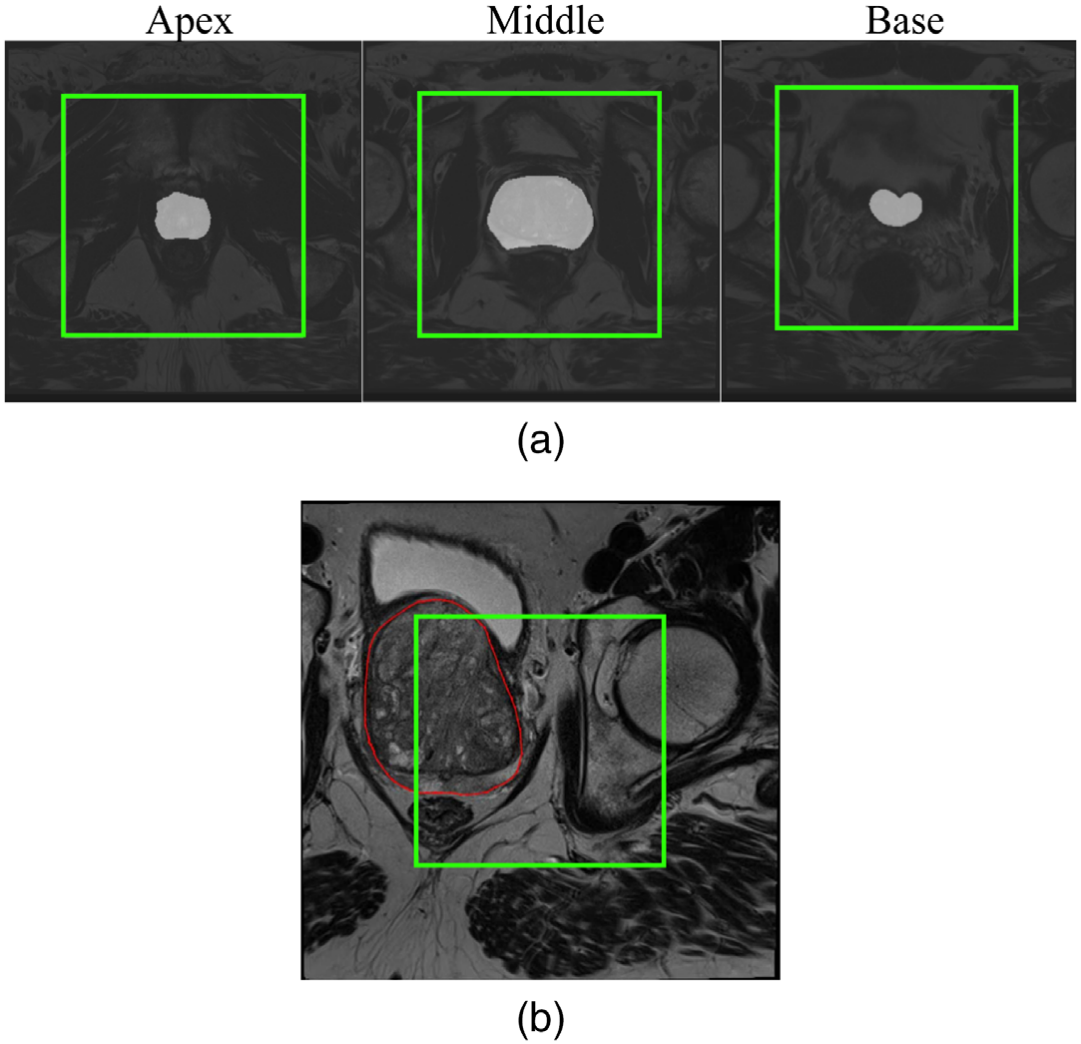
(a) Prostate slices from the apex, middle, and base of a patient with an original image size of 384×384. The slices have a pixel spacing of 0.5  mm×0.5  mm, and the cropped square box (256×256, in green) is centered on the midpoint of the prostate. The foreground and background pixels are unbalanced, resulting in an image that contains little information about the prostate. (b) A middle slice from a patient with benign prostatic hyperplasia, in which cropping to the center of the image fails. The area surrounded by red represents the segmented prostate gland. Image size is 384×384, pixel spacing is 0.5  mm×0.5  mm, and cropped area is 256×256. The image was acquired at St. Olavs Hospital, Trondheim University Hospital, Norway. Use was approved by the institutional review board and The Regional Committee for Medical and Health Research Ethics (REC Central Norway, identifier 2017/576, 2013/1869).

For PCa, due to input constraints, it is challenging for most deep learning models to obtain an image that contains the entire prostate with a balanced pixel distribution, as shown in Fig. 1(a). One solution is to crop the image to the region containing the ROI to achieve a better balance between foreground and background pixels. ROIs can be cropped manually or automatically. Manual cropping is a tedious and time-consuming task. Therefore, automated cropping methods are more commonly used for tasks that require large numbers of images, such as training deep learning models. The most common approach is center cropping, which assumes that the ROI is located in the center of the image. However, due to differences in image acquisition protocols, there is a risk that the ROI will be cropped inaccurately, and the assumption that the prostate is always located in the center is not always true, as shown in Fig. 1(b).

A robust, flexible, and accurate tool for automated cropping of ROIs, which is compatible with deep learning algorithms and can capture balanced foreground and background pixels in a standardized manner, is currently lacking. Consequently, the contributions of this work are (1) the introduction of CROPro, an open source, publically available tool for standardizing the automated cropping of ROIs regardless of patient health status, ROI size, image size, or pixel spacing and (2) to demonstrate its use by evaluating the impact of image cropping settings in the context of classification of csPCa on T2-weighted (T2W) MR images with CNN and ViT-based models.

## 2 Related Work

Recently, several deep learning-based approaches have been developed for PCa detection and classification,^9^^,^^23^^,^^29^^–^^36^ and the cropping strategy varied between papers. Wang et al.^33^ trained a deep CNN to discriminate PCa patients from benign prostate conditions. T2W MR images from 172 patients were used. Each image was downsized to 360×360  pixels and then cropped into multiple subimages of 288×288  pixels, resulting in a area under the receiver operating characteristic curve (AUC) of 0.84. Yoo et al.^9^ proposed a two-level (slice and patient level) automated deep CNN-based pipeline to detect csPCa. Diffusion-weighted MR images (DWI) from 427 patients were used as the dataset. Each DWI slice was resized to a fixed size of 144×144 and then center cropped to 66×66  pixels, resulting in an AUC of 0.87 and 0.84 at slice and patient level, respectively. Vente at al.^29^ used 2D U-Net to both detect and identify the Gleason grade group to estimate lesion aggressiveness on the PROSTATEx-2 challenge dataset. This resulted in a lesion-based weighted kappa of 0.13±0.27. Cropped ROIs of 90  mm×90  mm×80  mm, with visual verification of the prostate gland presence within the cropped images were resized to 192×192×32  voxels for training. Yang et al.^34^ developed a deep CNN for PCa detection using mpMRI from 780 patients. They cropped the prostate gland using the prostate segmentation mask with a fixed bounding box of 196×196×16  voxels and spacing of 0.4  mm×0.4  mm×0.5  mm and achieved an AUC of 0.96. Saha et al.^35^ proposed two parallel 3D CNNs for patient-level detection of csPCa with an AUC of 0.882±0.030 trained on 1584 MRI scans and tested on 486 scans with PI-RADS v2 annotation, with an input ROI of 144×144×18  voxels for the detection model and 112×112×12  voxels for a residual classifier. In their study, the residual classifier takes multichannel batches of 64×64×8  voxels with a stride of 16 (in-plane) and 4 voxels (through-plane) as input to generate a malignant score per image patch, which is fused with the detection model to identify csPCa. 296 scans with external biopsy confirmation were used to test agreement between model, radiologists (kappa=0.51±0.4) and pathologists (kappa=0.56±0.6). The input was resampled to a common resolution of 0.5  mm×0.5  mm×3.6  mm. Hosseinzadeh at al.^36^ used U-Net for detection and localization of csPCa (PI-RADS ≥4) with an AUC of 0.88, where input images were resampled to 0.5  mm×0.5  mm and then cropped by 9.6×9.6  cm around the center. A dataset of 2734 mpMRI scans was used for training and testing.

Pachetti et al.^23^ evaluated ViTs for classification low-grade versus high-grade PCa lesions (Gleason score ≤3+4 versus ≥4+3) trained from scratch on ProstateX-2 with axial volumetric T2W images, with a mean AUC of 0.775±0.094 on fivefold cross validation. All images were scaled to the largest image size in the dataset (384×384) and cropped with a fixed size of 128×128, assuming that the prostate was located in the center of the image. Lesion volumes of size 128×128×5 were used for analysis. In summary, the size of the input images to the models reported above varied and none of the studies reported optimization of the cropping strategy.

An automated tool for standardized cropping of prostate images would presumably help increase model robustness, comparability, and generalizability between centers. In their PCa detection system, Yang et al.^37^ proposed to automatically crop the image using a regression CNN model to prune a square region containing the entire prostate gland. For this purpose, T2W original images were paired with manually labeled square bounding boxes. Although the subsequent step in their detection network can handle varying image sizes resulting from this approach, it is limited to the size of the manual square bounding boxes and does not take into account pixel spacing, which can vary from patient to patient and protocol to protocol. Zaridis et al.^38^ proposed an automated prostate gland cropping approach based on a U-Net to predict an amorphous region around the prostate. A bounding box was determined using the minimum and maximum coordinates of the amorphous mask and resampled to 256×256  pixels. This approach improved the deep learning-based segmentation accuracy with up to 8.9%. However, resampling an image to a fixed size will cause the image to be scaled and stretched, which may affect model performance since the original image is modified.

## 3 Proposed Method

There is a need for an automated cropping tool for deep learning applications that works under the following conditions:

• *Image size*. The tool can handle arbitrary-sized images as input and return cropped images of chosen size as output.
• *Image resolution*. The tool can handle images with any resolution (pixel spacing) as input and return cropped images with chosen resolution as output.
• *Coverage*. The tool offers different sampling strategies to cover the complete ROI.

Meeting these conditions enables flexible image sampling, compatible with any deep learning method and allows optimizing the balance between foreground and background pixels for the problem at hand.

**Fig. 2:**
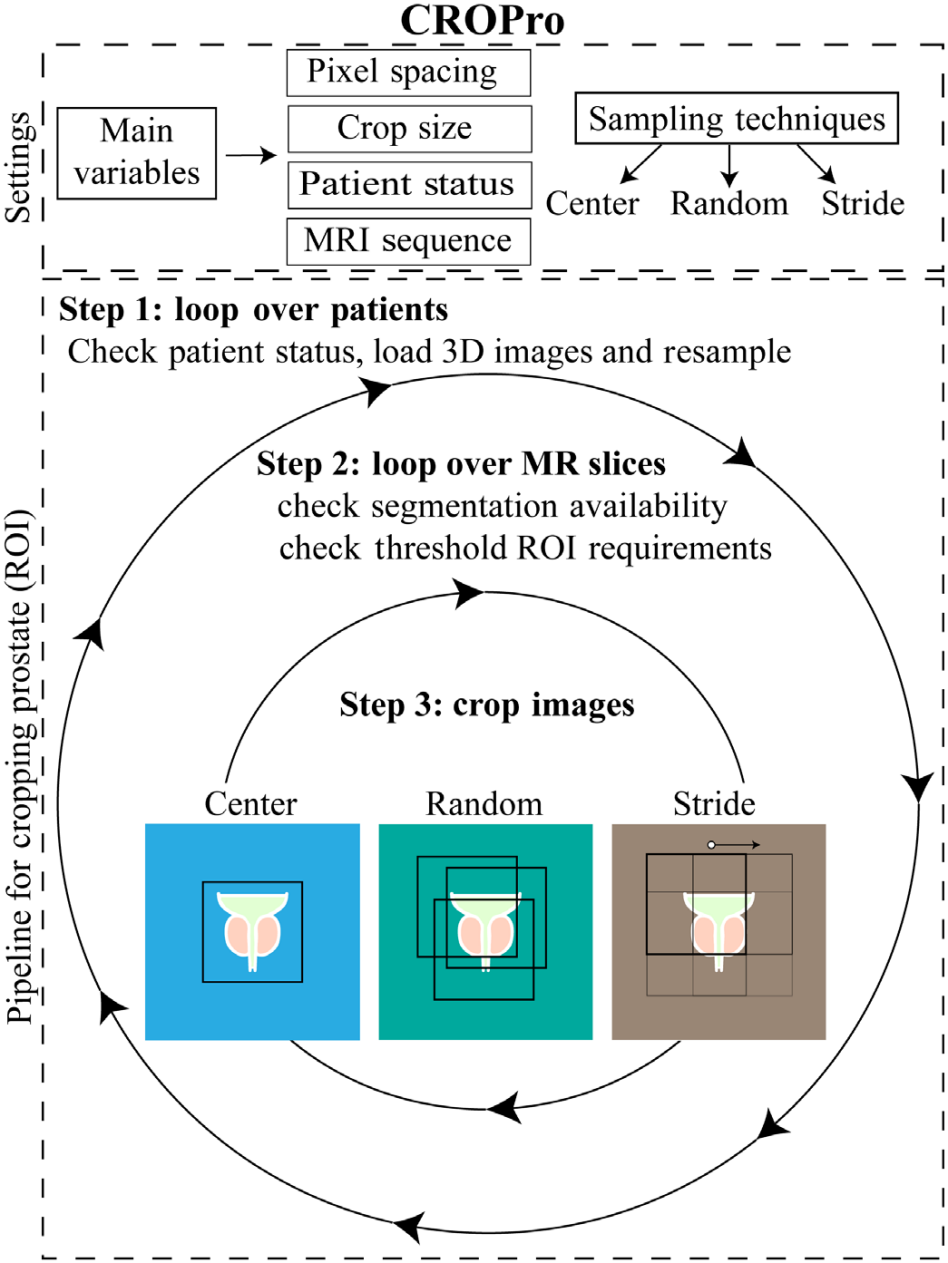
The pipeline of the CROPro tool. Among the different settings, the pixel spacing, the size of the cropped image, and three different sampling techniques can be freely selected, along with several other settings, such as cropping factors depending on the selected sampling technique, the patient’s health status, the type of MRI sequence, and the type of image to be stored. Depending on the patient’s health status regarding prostate cancer (positive, negative, or unknown), a loop is started for all MRI slices, checking the availability of a segmentation mask and the fulfillment of threshold ROI size criteria (i.e., cropped area > minimum ROI area) before applying the cropping techniques.

CROPro, our proposed tool, can crop images of any size and resolution using three sampling strategies: center cropping, random cropping, and stride cropping. Each of these strategies is based on a (manually or automatically) segmented ROI, e.g., the whole prostate gland or a suspected lesion. The general method is illustrated in Fig. 2 and outlined in detail in the next sections.

### 3.1 Image Resampling

**Fig. 3:**
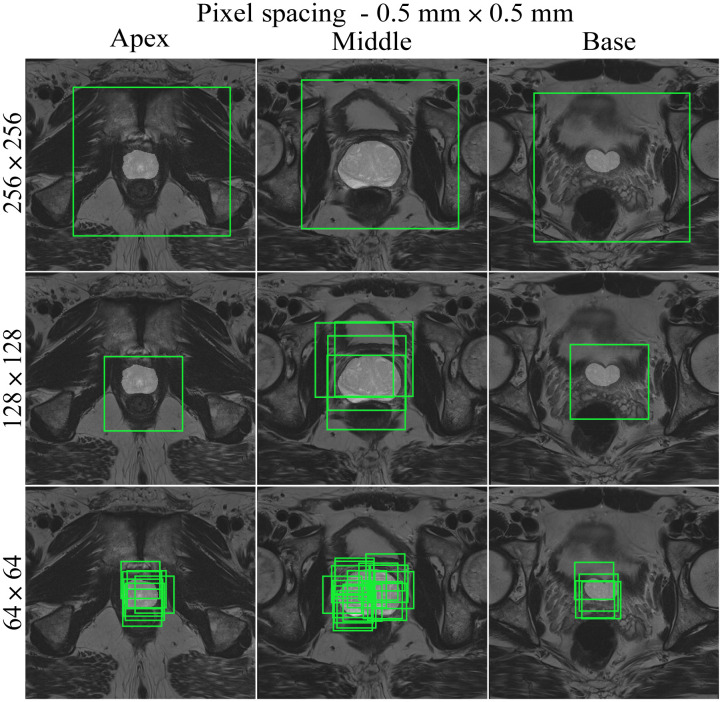
Slices through the apex, middle, and base of the prostate for a negative patient, using the random cropping function. Three different cropped image sizes (64×64, 128×128, and 256×256) are shown, overlaid with cropping boxes in light green color. The images have 0.5  mm×0.5  mm pixel spacing.

**Fig. 4:**
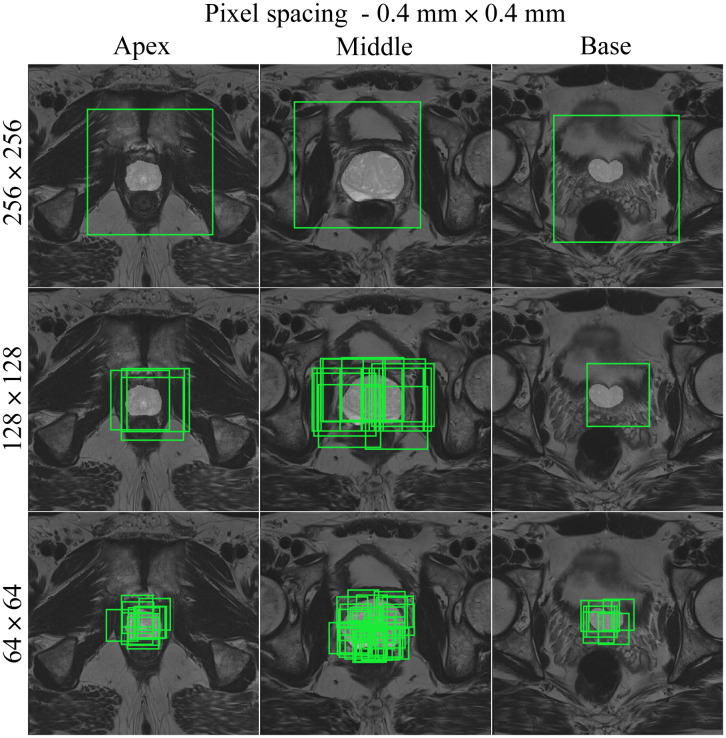
Slices through the apex, middle, and base of the prostate for a negative patient, using the random cropping function. Three different cropped image sizes (64×64, 128×128, and 256×256) are shown, overlaid with cropping boxes in light green color. The images have 0.4  mm×0.4  mm pixel spacing.

**Fig. 5:**
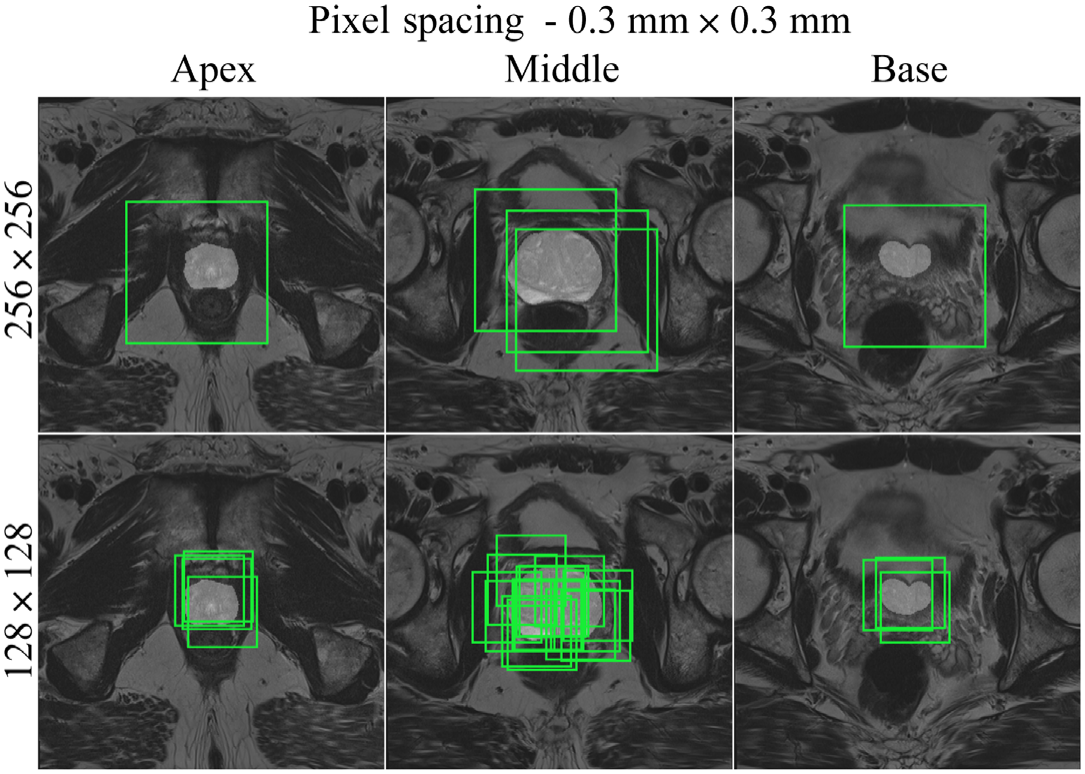
Slices through the apex, middle, and base of the prostate for a negative patient, using the random cropping function. Two different cropped image sizes (128×128 and 256×256) are shown, overlaid with cropping boxes in light green color. The images have 0.3  mm×0.3  mm pixel spacing.

**Fig. 6:**
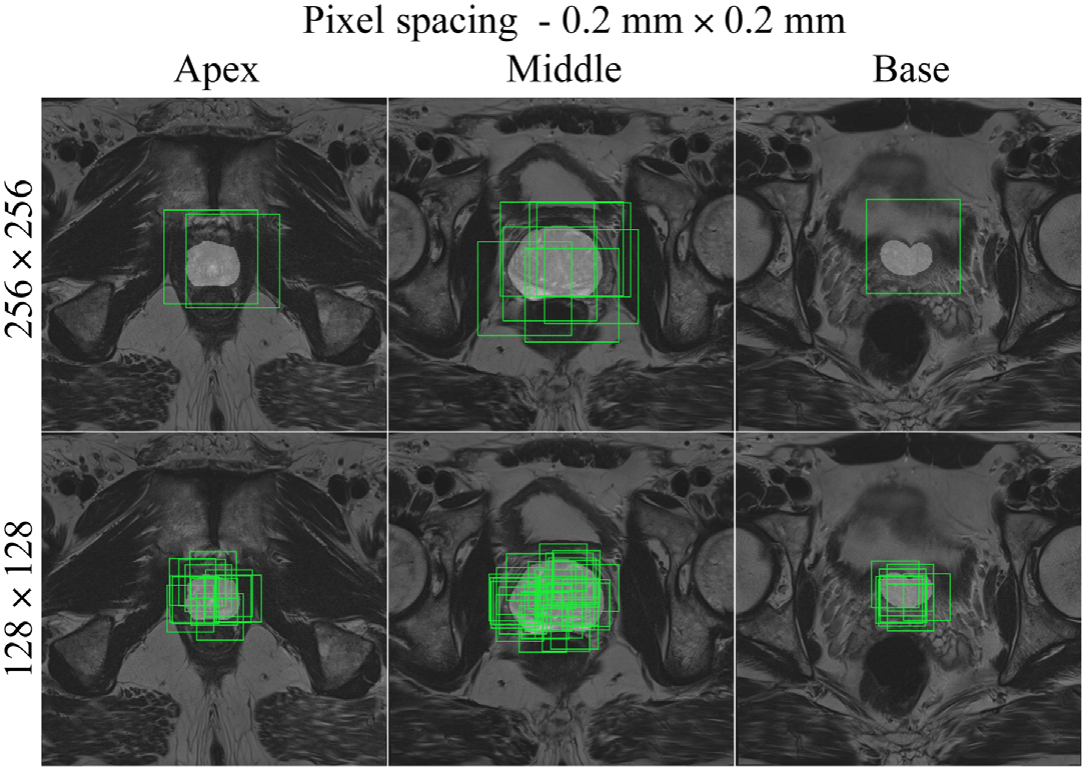
Slices through the apex, middle, and base of the prostate for a negative patient, using the random cropping function. Two different cropped image sizes (128×128 and 256×256) are shown, overlaid with cropping boxes in light green color. The images have 0.2  mm×0.2  mm pixel spacing.

Medical images are acquired at different institutions with varying scanners and slightly different scanning protocols. Consequently, the image resolution or pixel spacing (measured in mm) of the input images in a dataset may be different. This is not ideal for deep learning purposes as the model performance can be affected if a trained model is tested on data with a different resolution. Resampling the images to the same pixel spacing helps solving this problem. In addition, adjusting the pixel spacing in combination with the crop size can help optimize the balance between foreground and background pixels. Figures 3–6 show different pixel spacings and crop sizes based on the segmentation mask. For example, images of size 128×128  pixels with pixel spacing of 0.5  mm×0.5  mm (Fig. 3) have more background pixels than images of size 128×128  pixels with a pixel spacing of 0.4  mm×0.4  mm (Fig. 4).

CROPro allows resampling of images with different pixel spacings. The mask image (segmentation) is interpolated using nearest neighbor interpolation, and B-spline interpolation is used to convert the original images into the new pixel space. Both techniques are commonly used for resampling of medical images.^20^ The resampling is performed using SimpleITK (Python version 1.2.0).^39^

### 3.2 Image Cropping

CROPro can crop input images to output images of any chosen size with three different sampling techniques (center, random, and stride). In center cropping, a single output image of chosen size is sampled from the center of the segmented ROI. In random cropping, one or more output images of chosen size are sampled from the ROI by setting the center of the cropping mask to a random pixel in the ROI. In CROPro, the number of randomly cropped samples ($N_{\text{samples}}$) is controlled by the parameter $C_{\text{random}}$ in the following equation:

$$N_{\text{samples}}=\frac{N_{\mathrm{roi}}}{N_{\text{crop}}}*C_{\text{random}},$$ (1)

where $N_{\mathrm{roi}}$ is the number of pixels in the ROI and $N_{\text{crop}}$ is the number of pixels in the cropped output images, e.g., 64×64, 128×128, or 256×256. $C_{\text{random}}$ is an empirically chosen factor that controls the number of samples required to cover the ROI and is only used in random cropping mode. Setting $C_{\text{random}}$ too low could result in undersampling of the ROI, whereas too high values of $C_{\text{random}}$ could result in oversampling. For example, with $N_{\mathrm{roi}}=10,000$ and $N_{\text{crop}}=128\times 128$, the division in Eq. (1) is equal to 0.610. Setting $C_{\text{random}}$ to 10 will then result in $N_{\text{samples}}$ equal to 6.10. $N_{\text{samples}}$ is rounded down to the nearest integer, resulting in six samples in this example. Of note, the choice of $C_{\text{random}}$ is not affected by pixel spacing, ROI size, and crop size, as $N_{\text{samples}}$ scales automatically with these parameters.

In stride cropping, one or more output images of chosen size are sampled from a rectangular box around the ROI by systematically moving from top left to bottom right, skipping $C_{\text{stride}}$ pixels in each direction. Consequently, the number of samples is given by the following equation:

$$N_{\text{samples}}=\frac{H_{\text{box}}-H_{\mathrm{im}}}{N_{\text{stride}+1}}*\frac{W_{\text{box}}-W_{\mathrm{im}}}{N_{\text{stride}+1}},$$ (2)

where $H_{\text{box}}$ and $W_{\text{box}}$ are the height and width, respectively, of a box covering the entire ROI rounded up to the nearest multiple of $C_{\text{stride}}$, and $H_{\mathrm{im}}$ and $W_{\mathrm{im}}$ are the chosen height and width of the output image.

### 3.3 Image Selection

To present the deep learning network with only the information relevant to the task at hand, we often want to select the images that contain a significant part of the ROI. Images that have limited task-related information, e.g., containing only a very small part of a lesion, could potentially harm the training procedure. To ensure that images with too little task-related information are not included in the training set, we introduce a threshold parameter $C_{\min\_\text{area}}$, which specifies the minimum area (in ${\mathrm{mm}}^{2}$) of ROI that needs to be present in a cropped image. The value of $C_{\min\_\text{area}}$ depends on the clinical problem and needs to be determined and adjusted per use case. In CROPro, a cropped image will only be processed if Eq. (3) is true:

$$C_{\min\_\text{area}}<N_{\mathrm{roi}}*{\text{pixel}\_\text{spacing}}^{2}.$$ (3)

## 4 Experiments and Results

The Ubuntu 18.04.5 LTS operating system with a single NVIDIA Tesla V100S PCIe 32 GB GPU was used for all experiments. Using transfer learning, five CNN-based models and five ViT-based models^13^ were trained, validated, and tested for image-level csPCa classification. Our hypothesis is that the performance of the models depends on the CROPro settings for cropping of the input images. The code is written in Python (version 3.6.9).

### 4.1 Dataset

The publicly available PI-CAI challenge dataset^40^ (N=1500) was used to train, validate, and test the 10 deep learning models. 25 cases were excluded, due to having more than one scan per patient (N=24) or lacking clinical information (N=1). The cases were classified as positive (N=425) or negative (N=1050) for csPCa based on International Society of Urological Pathology criteria for grading of PCa.^41^ The dataset was divided into a training set (70%, N=1033, 736 negative and 297 positive cases), validation set (15%, N=221, 157 negative and 64 positive cases), and a test set (15%, N = 221, 157 negative and 64 positive cases). The split was done randomly for both negative and positive cases, with the exception that the validation and test sets were assigned only positive cases with human labels. In contrast, the training set contained a mix of human (N=92) and AI-labeled (N=205) positive cases. The T2W MR images were normalized using AutoRef ^42^ and used as input for the networks. The pixel spacing of the original images varied from 0.23  mm×0.23  mm to 0.78  mm×0.78  mm, and interslice spacing ranged from 2.2 to 5.0 mm. Image size varied from 256×256 to 1024×1024. In this study, PI-CAI data version 1.0 was used for all experiments.

### 4.2 CROPro Parameters

**Fig. 7:**
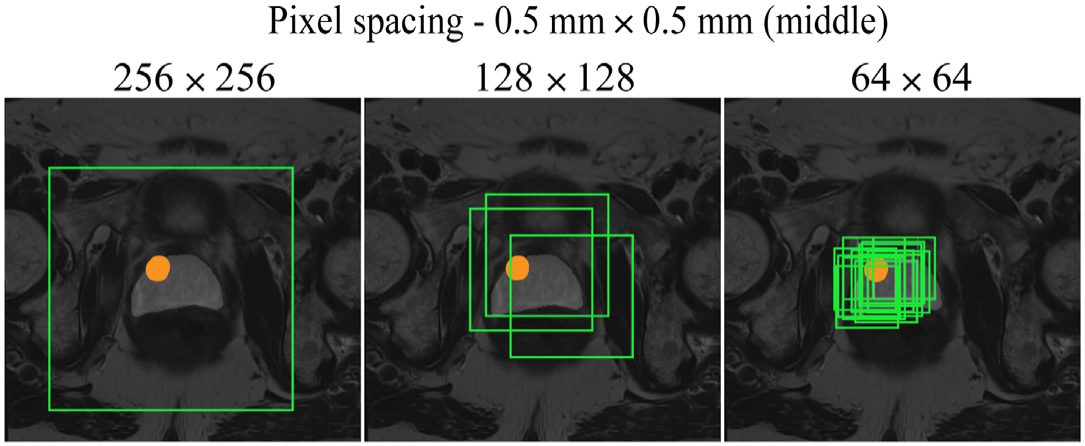
A slice through the middle of the prostate for a positive patient using the random cropping technique. Three different cropped image sizes (64×64, 128×128, and 256×256) are shown, overlaid with the cropping boxes in light green color. The orange area represents the lesion delineation. The images have 0.5  mm×0.5  mm pixel spacing.

The following CROPro settings were investigated for cropping the T2W MR images in the training set: pixel spacing 0.2  mm×0.2  mm, 0.3  mm×0.3  mm, 0.4  mm×0.4  mm, and 0.5  mm×0.5  mm; image size 64×64, 128×128, and 256×256  pixels; and sampling with center cropping, random cropping, and stride cropping. $C_{\text{random}}$ was empirically set to 12 and $C_{\text{stride}}$ to 32. $C_{\min\_\text{area}}$ was set to $1\;{\mathrm{cm}}^{2}$ (prostate mask) for negative cases, and to $0.2\;{\mathrm{cm}}^{2}$ (lesion mask) for positive cases. This means that only cropped images containing more than $1\;{\mathrm{cm}}^{2}$ of the prostate ROI were used for the negative cases, and cropped images with more than $0.2\;{\mathrm{cm}}^{2}$ of lesion ROI were used for the positive cases. Segmentations of the first (apex) and last (base) slices of the prostate gland were excluded to avoid bias in AI-based segmentation. Furthermore, segmented lesions were retained only if they overlapped with ≥50% of the prostate mask. During validation and testing, images were systematically sampled using stride cropping (with the same $C_{\text{stride}}$ and $C_{\min\_\text{area}}$) to ensure that the entire prostate ROI was covered. Table 1 provides information on the datasets generated with the investigated CROPro settings. The combinations of image size 64×64 and pixel spacing of 0.3  mm×0.3  mm and 0.2  mm×0.2  mm were considered to provide too small images and were excluded from further analysis. Figures 3–6 show three slices (apex, middle, and base) from a selected negative patient using the random cropping technique with different image sizes and different values for pixel spacings. Figure 7 shows an example of a positive patient for a pixel spacing of 0.5  mm×0.5  mm and different cropped image sizes. The number of sampled images increases for smaller cropped image sizes, smaller pixel spacings, and larger prostate ROIs.

**Table 1:** Details about all datasets generated using center, random, and stride cropping with three different cropped sizes (64×64, 128×128, and 256×256) and four different pixels spacings (0.5  mm×0.5  mm, 0.4  mm×0.4  mm, 0.3  mm×0.3  mm, and 0.2  mm×0.2  mm).

Cropped image (pixels^2^)	Pixel space (${\mathrm{mm}}^{2}$)	Train center neg. (N=736)/pos. (N=297)	Train random neg. (N=736)/pos. (N=297)	Train stride neg. (N=736)/pos. (N=297)	Val. stride neg. (N=157)/pos. (N=64)	Test stride neg. (N=157)/pos. (N=64)
256 × 256	0.5 × 0.5	9143/1069	9322/1058	9145/1069	1891/265	1940/288
256 × 256	0.4 × 0.4	9183/1088	12,327/1220	9183/1089	1917/275	1945/319
256 × 256	0.3 × 0.3	9156/1091	22,731/2221	9218/1091	1920/274	1965/320
256 × 256	0.2 × 0.2	5796/843	55,945/5616	18,711/1407	3970/388	4563/537
128 × 128	0.5 × 0.5	8595/1086	34,290/3408	9873/1102	2066/279	2132/331
128 × 128	0.4 × 0.4	5712/826	55,946/5613	15,267/1324	3254/343	3456/458
128 × 128	0.3 × 0.3	1917/212	102,937/10,429	41,719/2667	9059/725	9513/943
128 × 128	0.2 × 0.2	276/16	237,376/24,131	167,816/7489	35,902/2008	37,575/2781
64 × 64	0.5 × 0.5	816/70	150,304/15,255	44,407/2454	9483/664	9783/838
64 × 64	0.4 × 0.4	256/15	237,392/24,118	75,527/3412	16,089/900	16,566/1187

### 4.3 csPCa Classification with CNNs and ViTs

AlexNet,^43^ VGG-19,^44^ ResNet50,^8^ SqueezeNet,^45^ and DesnseNet121^46^ and five ViT^13^ models were trained, validated, and tested for image-level classification of csPCa. Each ViT model represents a different combination of model size (base, large, and huge)^13^ and patch size (14×14, 16×16, and 32×32). Table 2 provides an overview of all deep learning models used in this study. We investigated the impact of the different CROPro settings on model performance.

**Table 2:** An overview of the CNN and ViT-based models used for classification of csPCa. For the ViT models, each model represent a variant of the initial ViT model^13^ in terms of model size (parameters) and input patch size (14×14, 16×16, and 32×32). The CNN models were loaded with code from github.com/pytorch. All CNN models can be found here: pytorch.org/vision. The ViT models were loaded with code from github.com/huggingface/transformers. All available ViT models can be found here: huggingface.co/models.

CNN models	ViT models
AlexNet^43^	ViT-H/14 (https://huggingface.co/google/vit-huge-patch14-224-in21k)
VGG-19^44^	ViT-L/32 (https://huggingface.co/google/vit-large-patch32-224-in21k)
ResNet50^8^	ViT-L/16 (https://huggingface.co/google/vit-large-patch16-224-in21k)
SqueezeNet^45^	ViT-B/32 (https://huggingface.co/google/vit-base-patch32-224-in21k)
DesnseNet121^46^	ViT-B/16 (https://huggingface.co/google/vit-base-patch16-224-in21k)

Overall, 30 different training datasets were investigated for each model, leading to 300 combinations. Each model was trained 5 times for each combination. The cases in the training, validation, and test sets were kept the same to allow for fair comparison between experiments. Each generated dataset was balanced by randomly selecting the same number of negative images as the available number of positive images.

The deep learning models were pretrained with ImageNet^47^ on ImageNet-1k (CNNs) and ImageNet-21k (ViTs). Transfer learning of the CNNs was implemented using feature extraction, where only the weights of the last layer related to the prediction were updated. Hyperparameters were the same for all models and experiments: batch size 64; the number of epochs 100 with early stopping and a patience count of 10; Adam optimizer with learning rate 0.001, beta1 0.9 and beta2 0.999. The ViT models were implemented using huggingface transformers library.^48^ Hyperparameters were the same for all ViT models and experiments using the class trainer API provided by huggingface: batch size 30, epochs 5, learning rate 0.0002, evaluation strategy with steps, fp16 bit precision, save steps 100, evaluation steps 100, and logging steps 10. PyTorch (version 1.9.1) python library^49^ was used for implementation.

### 4.4 Statistical Analysis

Classification accuracy was used as a metric of performance. The mean and standard deviation (SD) of 5 runs for each model on the validation and test sets were reported. The performance of each model was compared to the reference model with image size 256×256 and 0.5  mm×0.5  mm pixel spacing. Statistical differences were assessed using two-sample paired t-tests. P-values<0.05 were considered statistically significant.

### 4.5 Results

In the following section, the results from the test set are presented separately for each of the sampling techniques. The results from the validation set are provided to the reader as Supplementary Material.

#### 4.5.1 Center cropping

**Fig. 8:**
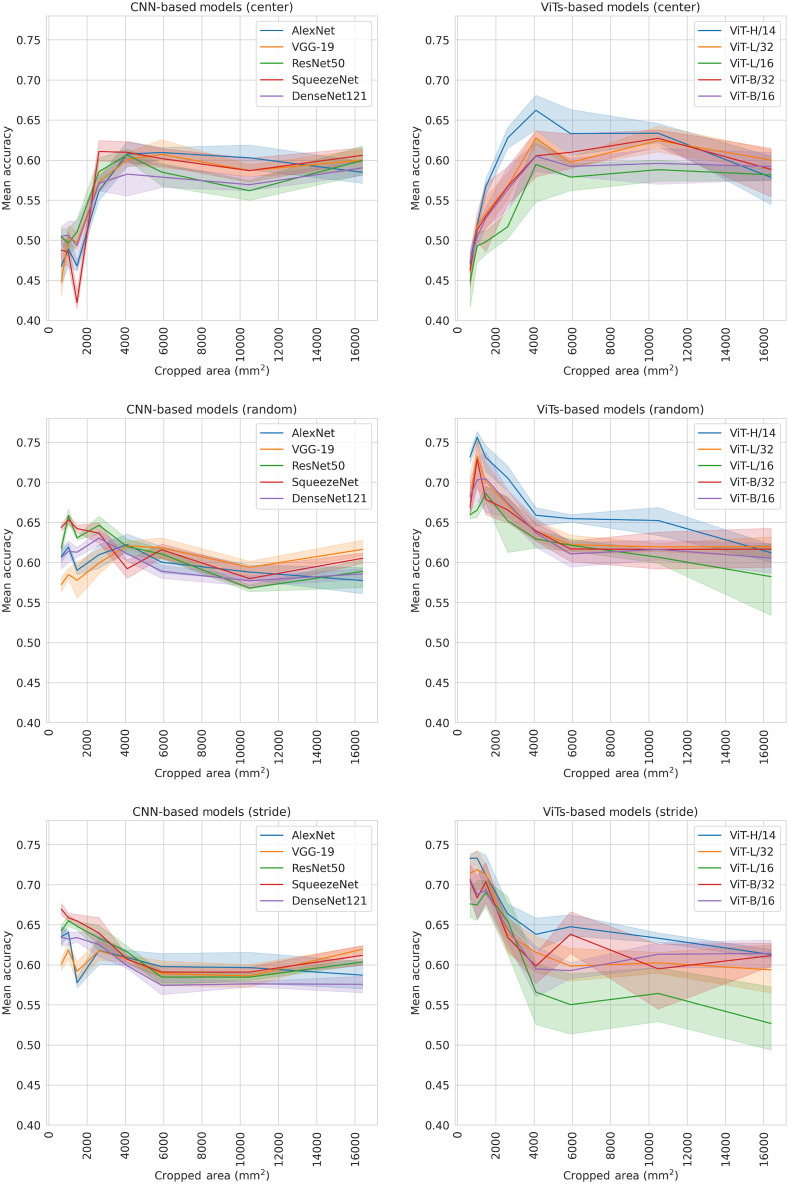
The performance of each sampling technique (center, random, and stride) for all CNN and ViT-based models, as a function of the area of the cropped images. The solid lines represent the mean accuracy and the shaded areas the 95% confidence intervals.

Table 3 shows the mean ± SD for all CNN-based models and Table 4 for all ViT-based models. Both tables represent different settings with center cropping. For CNN-based models, the highest performance (0.621±0.022) was obtained by SqueezeNet with a cropped image size of 256×256 and a pixel spacing of 0.2  mm×0.2  mm. For each network, the model with the best performance was compared with the reference model. Significant improvements were found for AlexNet and SqueezeNet, but not for VGG-19, ResNet50, and DenseNet121 when using either a smaller cropped image size or a smaller pixel spacing (Fig. 8). For ViT-based models, the highest performance (0.662±0.028) was obtained by ViT-H/14 with a cropped image size of 128×128 and a pixel spacing of 0.5  mm×0.5  mm. The best models performed significantly better than the reference model for ViT-H/14, ViT-L/32 but not for ViT-L/16, ViT-B/32, and ViT-B/16.

**Table 3:** The test accuracy of the five convolutional neural network models for different CROPro settings with center cropping. The best performing settings are highlighted in bold and significant differences between these models and the reference model are indicated with a “*”.

Cropped image (${\text{pixels}}^{2}$)	Pixel space (${\mathrm{mm}}^{2}$)	AlexNet	VGG19	ResNet50	SqueezeNet	DenseNet121
256 × 256	0.5 × 0.5	0.584 ± 0.017	0.600 ± 0.017	0.599 ± 0.023	0.605 ± 0.010	**0.590 ± 0.012**
256 × 256	0.4 × 0.4	0.602 ± 0.019	0.586 ± 0.009	0.561 ± 0.015	0.586 ± 0.018	0.569 ± 0.010
256 × 256	0.3 × 0.3	0.609 ± 0.009*	**0.607 ± 0.021**	0.585 ± 0.019	0.601 ± 0.013	0.578 ± 0.017
256 × 256	0.2 × 0.2	0.570 ± 0.035	0.564 ± 0.022	0.594 ± 0.030	0.621 ± 0.022*	0.572 ± 0.021
128 × 128	0.5 × 0.5	0.607 ± 0.021	0.600 ± 0.011	**0.606 ± 0.008**	0.609 ± 0.017	0.582 ± 0.033
128 × 128	0.4 × 0.4	0.552 ± 0.007	0.580 ± 0.021	0.576 ± 0.027	0.599 ± 0.016	0.569 ± 0.012
128 × 128	0.3 × 0.3	0.468 ± 0.008	0.497 ± 0.013	0.510 ± 0.019	0.422 ± 0.016	0.493 ± 0.041
128 × 128	0.2 × 0.2	0.438 ± 0.029	0.436 ± 0.039	0.499 ± 0.014	0.478 ± 0.038	0.517 ± 0.018
64 × 64	0.5 × 0.5	0.488 ± 0.028	0.503 ± 0.015	0.496 ± 0.023	0.484 ± 0.018	0.506 ± 0.020
64 × 64	0.4 × 0.4	0.496 ± 0.004	0.459 ± 0.025	0.508 ± 0.019	0.497 ± 0.032	0.492 ± 0.016

**Table 4:** The test accuracy of the five vision transformer models for different CROPro settings with center cropping. The best performing settings are highlighted in bold and significant differences between these models and the reference model are indicated with a “*”.

Cropped image (${\text{pixels}}^{2}$)	Pixel space (${\mathrm{mm}}^{2}$)	ViT-H/14	ViT-L/32	ViT-L/16	ViT-B/32	ViT-B/16
256 × 256	0.5 × 0.5	0.578 ± 0.036	0.600 ± 0.016	0.581 ± 0.008	0.588 ± 0.040	0.592 ± 0.020
256 × 256	0.4 × 0.4	0.633 ± 0.015	0.624 ± 0.021	0.588 ± 0.015	**0.627 ± 0.013**	0.595 ± 0.028
256 × 256	0.3 × 0.3	0.633 ± 0.036	0.597 ± 0.007	0.578 ± 0.021	0.609 ± 0.029	0.592 ± 0.013
256 × 256	0.2 × 0.2	0.628 ± 0.029	0.557 ± 0.035	0.502 ± 0.005	0.573 ± 0.028	0.566 ± 0.055
128 × 128	0.5 × 0.5	0.662 ± 0.028*	0.627 ± 0.010*	**0.594 ± 0.055**	0.605 ± 0.038	**0.604 ± 0.021**
128 × 128	0.4 × 0.4	0.627 ± 0.010	0.577 ± 0.020	0.531 ± 0.036	0.560 ± 0.050	0.559 ± 0.040
128 × 128	0.3 × 0.3	0.566 ± 0.013	0.533 ± 0.027	0.497 ± 0.017	0.529 ± 0.053	0.527 ± 0.015
128 × 128	0.2 × 0.2	0.481 ± 0.018	0.459 ± 0.032	0.427 ± 0.060	0.455 ± 0.043	0.465 ± 0.026
64 × 64	0.5 × 0.5	0.518 ± 0.007	0.517 ± 0.013	0.492 ± 0.024	0.513 ± 0.024	0.504 ± 0.016
64 × 64	0.4 × 0.4	0.462 ± 0.010	0.461 ± 0.027	0.471 ± 0.025	0.471 ± 0.030	0.480 ± 0.022

#### 4.5.2 Random cropping

Tables 5 (CNN-based models) and 6 (ViT-based models) show the mean ± SD for all trained models and different settings with random cropping. For CNNs, the highest performance (0.662±0.005) was achieved by ResNet50 with a cropped image size of 256×256 and pixel spacing of 0.2  mm×0.2  mm. The best models performed significantly better than the reference model for all networks except VGG19, when using either a smaller cropped image size or a smaller pixel spacing (Fig. 8). The best models with random cropping performed significantly better than the best models with center cropping for all models except AlexNet and VGG19. For ViTs, the highest performance (0.756±0.009) was obtained by ViT-H/14 with a cropped image size of 64×64 and a pixel spacing of 0.5  mm×0.5  mm. The best models performed significantly better than the reference model for all models. Random cropping performed significantly better than center cropping for all models.

**Table 5:** The test accuracy of the five convolutional neural network models for different CROPro settings with random cropping. The best performing settings are highlighted in bold and significant differences between these models and the reference model are indicated with a “*”.

Cropped image (${\text{pixels}}^{2}$)	Pixel space (${\mathrm{mm}}^{2}$)	AlexNet	VGG19	ResNet 50	SqueezeNet	DenseNet 121
256 × 256	0.5 × 0.5	0.577 ± 0.021	0.616 ± 0.013	0.588 ± 0.023	0.605 ± 0.008	0.585 ± 0.008
256 × 256	0.4 × 0.4	0.588 ± 0.011	0.594 ± 0.012	0.568 ± 0.005	0.579 ± 0.017	0.577 ± 0.007
256 × 256	0.3 × 0.3	0.600 ± 0.021	0.618 ± 0.016	0.610 ± 0.007	0.615 ± 0.011	0.589 ± 0.010
256 × 256	0.2 × 0.2	0.611 ± 0.034	0.607 ± 0.034	0.662 ± 0.005*	0.650 ± 0.019	0.630 ± 0.018*
128 × 128	0.5 × 0.5	0.622 ± 0.014*	**0.620 ± 0.006**	0.619 ± 0.013	0.592 ± 0.015	0.610 ± 0.011
128 × 128	0.4 × 0.4	0.607 ± 0.013	0.591 ± 0.013	0.629 ± 0.011	0.622 ± 0.011	0.630 ± 0.012
128 × 128	0.3 × 0.3	0.590 ± 0.007	0.577 ± 0.028	0.630 ± 0.003	0.642 ± 0.005	0.612 ± 0.008
128 × 128	0.2 × 0.2	0.601 ± 0.018	0.568 ± 0.021	0.622 ± 0.013	0.650 ± 0.003	0.625 ± 0.016
64 × 64	0.5 × 0.5	0.619 ± 0.006	0.585 ± 0.008	0.658 ± 0.010	0.653 ± 0.008*	0.613 ± 0.017
64 × 64	0.4 × 0.4	0.605 ± 0.007	0.576 ± 0.007	0.613 ± 0.010	0.637 ± 0.003	0.587 ± 0.012

**Table 6:** The test accuracy of the five vision transformer models for different CROPro settings with random cropping. The best performing settings are highlighted in bold and significant differences between these models and the reference model are indicated with a “*”.

Cropped image (${\mathrm{pixels}}^{2}$)	Pixel space (${\mathrm{mm}}^{2}$)	ViT-H/14	ViT-L/32	ViT-L/16	ViT-B/32	ViT-B/16
256 × 256	0.5 × 0.5	0.612 ± 0.014	0.619 ± 0.016	0.582 ± 0.056	0.616 ± 0.030	0.605 ± 0.023
256 × 256	0.4 × 0.4	0.652 ± 0.021	0.619 ± 0.006	0.606 ± 0.010	0.616 ± 0.029	0.616 ± 0.015
256 × 256	0.3 × 0.3	0.654 ± 0.006	0.622 ± 0.014	0.621 ± 0.009	0.616 ± 0.017	0.610 ± 0.019
256 × 256	0.2 × 0.2	0.710 ± 0.033	0.662 ± 0.019	0.653 ± 0.086	0.675 ± 0.016	0.669 ± 0.029
128 × 128	0.5 × 0.5	0.658 ± 0.015	0.638 ± 0.016	0.629 ± 0.014	0.639 ± 0.013	0.636 ± 0.023
128 × 128	0.4 × 0.4	0.700 ± 0.019	0.680 ± 0.028	0.650 ± 0.008	0.655 ± 0.031	0.678 ± 0.030
128 × 128	0.3 × 0.3	0.731 ± 0.020	0.700 ± 0.030	0.686 ± 0.013*	0.678 ± 0.023	0.704 ± 0.049*
128 × 128	0.2 × 0.2	0.734 ± 0.007	0.703 ± 0.045	0.663 ± 0.012	0.673 ± 0.015	0.693 ± 0.029
64 × 64	0.5 × 0.5	0.756 ± 0.009*	0.732 ± 0.042*	0.665 ± 0.011	0.728 ± 0.038*	0.703 ± 0.037
64 × 64	0.4 × 0.4	0.729 ± 0.015	0.662 ± 0.021	0.655 ± 0.005	0.664 ± 0.016	0.670 ± 0.038

#### 4.5.3 Stride cropping

Tables 7 (CNN-based models) and 8 (ViT-based models) show the mean ± SD for all trained models and different settings with stride cropping. For CNNs, the highest performance (0.678±0.006) was achieved by SqueezeNet with a cropped image size of 128×128 and a pixel spacing of 0.2  mm×0.2  mm. The best found models performed significantly better than the reference model when using either a smaller cropped image size or a smaller pixel spacing (Fig. 8) for all models except for VGG19. The best model with stride cropping performed significantly better than the best model with random cropping for AlexNet, DenseNet121, and SqueezeNet, but not for VGG19 and ResNet50. Furthermore, all models performed significantly better with stride cropping than with center cropping, except VGG19. For ViTs, the highest performance (0.741±0.022) was obtained by ViT-L/32 with a cropped image size of 128×128 and a pixel spacing of 0.2  mm×0.2  mm. The best models performed significantly better than the reference model for all models. The best models with stride cropping performed significantly better than the best models with center cropping, but not than random cropping, which performed significantly better for ViT-H/14.

**Table 7:** The test accuracy of the five convolutional neural network models for different CROPro settings with stride cropping. The best performing settings are highlighted in bold and significant differences between these models and the reference model are indicated with a “*”.

Cropped image (${\text{pixels}}^{2}$)	Pixel space (${\mathrm{mm}}^{2}$)	AlexNet	VGG19	ResNet 50	SqueezeNet	DenseNet121
256 × 256	0.5 × 0.5	0.587 ± 0.021	0.619 ± 0.006	0.603 ± 0.005	0.611 ± 0.017	0.575 ± 0.013
256 × 256	0.4 × 0.4	0.596 ± 0.025	0.586 ± 0.018	0.584 ± 0.005	0.590 ± 0.007	0.576 ± 0.004
256 × 256	0.3 × 0.3	0.597 ± 0.022	0.589 ± 0.020	0.584 ± 0.011	0.590 ± 0.007	0.574 ± 0.014
256 × 256	0.2 × 0.2	0.642 ± 0.021	**0.629 ± 0.022**	0.653 ± 0.017*	0.662 ± 0.024	0.642 ± 0.019
128 × 128	0.5 × 0.5	0.609 ± 0.012	0.606 ± 0.005	0.616 ± 0.009	0.606 ± 0.007	0.598 ± 0.005
128 × 128	0.4 × 0.4	0.593 ± 0.011	0.605 ± 0.011	0.612 ± 0.015	0.616 ± 0.011	0.607 ± 0.013
128 × 128	0.3 × 0.3	0.577 ± 0.007	0.591 ± 0.017	0.648 ± 0.006	0.654 ± 0.008	0.633 ± 0.008
128 × 128	0.2 × 0.2	0.647 ± 0.017*	0.605 ± 0.014	0.649 ± 0.012	0.678 ± 0.006*	0.650 ± 0.008*
64 × 64	0.5 × 0.5	0.640 ± 0.004	0.618 ± 0.013	0.655 ± 0.008	0.658 ± 0.006	0.632 ± 0.010
64 × 64	0.4 × 0.4	0.623 ± 0.011	0.595 ± 0.007	0.635 ± 0.004	0.659 ± 0.009	0.618 ± 0.008

**Table 8:** The test accuracy of the five vision transformer models for different CROPro settings with stride cropping. The best performing settings are highlighted in bold and significant differences between these models and the reference model are indicated with a “*”.

Cropped image (${\text{pixels}}^{2}$)	Pixel space (${\mathrm{mm}}^{2}$)	ViT-H/14	ViT-L/32	ViT-L/16	ViT-B/32	ViT-B/16
256 × 256	0.5 × 0.5	0.612 ± 0.007	0.593 ± 0.039	0.526 ± 0.051	0.611 ± 0.017	0.614 ± 0.022
256 × 256	0.4 × 0.4	0.633 ± 0.007	0.602 ± 0.018	0.564 ± 0.045	0.594 ± 0.054	0.613 ± 0.020
256 × 256	0.3 × 0.3	0.647 ± 0.016	0.598 ± 0.026	0.550 ± 0.047	0.638 ± 0.033	0.592 ± 0.013
256 × 256	0.2 × 0.2	0.665 ± 0.025	0.658 ± 0.018	0.678 ± 0.038	0.649 ± 0.026	0.656 ± 0.007
128 × 128	0.5 × 0.5	0.638 ± 0.028	0.615 ± 0.028	0.565 ± 0.049	0.598 ± 0.024	0.594 ± 0.041
128 × 128	0.4 × 0.4	0.661 ± 0.018	0.617 ± 0.040	0.630 ± 0.075	0.619 ± 0.012	0.633 ± 0.039
128 × 128	0.3 × 0.3	0.711 ± 0.033	0.712 ± 0.019	0.690 ± 0.021*	0.703 ± 0.032	0.693 ± 0.018
128 × 128	0.2 × 0.2	0.737 ± 0.010*	0.741 ± 0.022*	0.675 ± 0.009	0.714 ± 0.032*	0.735 ± 0.018*
64 × 64	0.5 × 0.5	0.733 ± 0.011	0.718 ± 0.031	0.674 ± 0.032	0.683 ± 0.036	0.687 ± 0.039
64 × 64	0.4 × 0.4	0.728 ± 0.008	0.687 ± 0.023	0.676 ± 0.043	0.694 ± 0.042	0.678 ± 0.025

## 5 Discussion

In this study, we introduced CROPro, an automated tool for standardized cropping of prostate MRIs to tackle challenges associated with manual preprocessing. We found that carefully optimizing the cropping of the prostate gland from the MR images improved csPCa classification with both CNN and ViT-based models. For both model types, the performance of the tested architectures showed similar dependencies on the cropped image size. For center cropping, the optimal performance was found for medium-sized images ($∼40\;{\mathrm{cm}}^{2}$). This corresponds to a cropped area that would cover the complete prostate in most images, but does not include too much of the surrounding tissue. For random and stride cropping, the optimal performance was generally associated with smaller images ($∼10\;{\mathrm{cm}}^{2}$). These would be large enough to cover most lesions while also capturing parts of the surrounding healthy tissue. VGG19 and AlexNet were exceptions to this behavior, but generally showed the lowest performance. It is important to stress that our results could have been different if yet other types of deep learning models, such as U-Net^28^ or GANs^50^ had been used, if the models had been trained from scratch, if a different classification task had been chosen (e.g., any PCa versus benign patients), and/or if different cropping settings had been tested.

All preprocessing steps for cropping images from the segmented prostate and lesion masks were automated and dependencies on ROI size, input image size and pixel spacing were eliminated. We used transfer learning with five pretrained CNN^51^ and five pretrained ViT^13^ models for all experiments as a faster alternative to training all models from scratch. This is a valid approach since we only aimed to investigate the impact of cropping parameters on model performance. Although we used state-of-the-art approaches to optimize the pretrained networks, the classification task could potentially benefit from further optimization of the hyperparameters and absolute model performance should therefore be evaluated in this context.

In our experiments, the stride count ($C_{\text{stride}}$) was set to 32 (stride cropping) and $C_{\text{random}}$ was set to 12 (random cropping) to balance under and oversampling of the ROI. $C_{\min\_\text{area}}$ was set to $0.2\;{\mathrm{cm}}^{2}$ for both AI- and human-derived lesion segmentations. The investigated crop sizes (256×256, 128×128, and $64\times 64\;{\text{ pixels}}^{2}$) and pixel spacings (0.2×0.2, 0.3×0.3, 0.4×0.4, and 0.5×0.5 mm^2^) cover a sensible range of values but could be set to any number.

For the CNN-based models, random and stride cropping generally outperformed center cropping, potentially due to the larger number of images available for training and/or a better ratio of foreground to background pixels. The best performance (0.678±0.006) was obtained with SqueezeNet trained on stride-sampled images of size 128×128 with a pixel spacing of 0.2  mm×0.2  mm. This was the highest image resolution tested and in accordance with findings in the literature^52^ that show a beneficial effect of higher resolution on classification performance.

For the ViT-based models, random and stride cropping generally outperformed center cropping, in line with results from the CNN-based models. The best performance for the ViT-based models was equal to 0.756±0.009 with ViT-H/14 trained on random-cropped images of size 64×64 with a pixel spacing of 0.5  mm×0.5  mm. For all cropping techniques, the best ViT-based model performed better than the best CNN-based model, confirming the potential of ViTs for image classification tasks.

In this study, we used both manual expert and AI segmentations of the prostate glands and lesions as input to CROPro. It should be noted that CROPro works with all types of segmentations, including those automatically generated by deep learning models, such as nnU-Net,^6^ which could reduce the workload of radiologists. This approach is in line with the proposal of Vente at al.,^28^ who mentioned that the selection of ROIs could be based on the segmentation of the prostate rather than capturing a fixed image center portion.

Several deep learning models have achieved high performance in classifying PCa^9^^,^^28^^,^^34^^,^^36^^,^^37^ but are dependent on input images of a specific size and pixel spacing. CROPro can be used as a simple tool to prepare the dataset for optimization and fair comparison of these models. In this regard, CAD systems, which have the potential to improve PCa detection, localization, staging, and biopsy targeting, can benefit from tools, such as CROPro to overcome challenges in automated analysis, such as selecting the correct ROI and dealing with input data that varies in pixel spacing and image dimensions.

Although cropping of the prostate appears to be a simple task, it can be quite challenging due to differences in image sources, the size of the prostate, and its location. For example, cropped images missing parts of the prostate may lead to misclassification, as 70% of PCa are located in the peripheral zone of the prostate.^53^ In addition, an automated tool should be adaptable and interpretable depending on the patient’s health status. For example, during training, it should avoid capturing slices from a positive patient that do not contain lesions. This is critical because these slices may be cancer-free (negative), meaning there is no useful information for classification. Unlike current approaches that focus on sectioning the entire prostate area,^9^^,^^28^^,^^33^^–^^35^^,^^37^^,^^38^ CROPro allows for the assignment of specific ROI types based on health status.

One limitation of our work was that we only tested CROPro with transfer-learned CNN-based and ViT-based models. Furthermore, only image-level accuracy for the test set was reported. Optimization of the csPCa classification task should be done with patient-level accuracy, but was considered outside the scope of this study. Another limitation was that we tested only 256×256, 128×128, and 64×64 input size images, whereas larger or smaller variations could have been considered. Moreover, the 3D MRI volumes were processed in such a way that cropped, 2D images were sampled and saved for each slice. Currently, CROPro does not take into account differences in through-plane resolution (slice thickness) between datasets. The implementation of cropping 3D volumes and handling different through-plane resolutions is subject of future work. Finally, our proposed CROPro tool was implemented and evaluated only for MRI and the task of csPCa classification. Evaluating the tool on other image modalities and/or clinical challenges would be interesting in the future.

## 6 Conclusion

We proposed and evaluated CROPro, a tool for automated cropping of prostate MRI to bypass manual data preprocessing and improve deep learning performance. We showed that the performance of the csPCa classification task depended on cropping parameters, indicating that fine-tuning these is important for reaching the full potential of deep learning applications.

## Data, Materials, and Code Availability

Code is available at: https://github.com/alexofficial/CROPro.
