# Supplementary material for: CROPro: a tool for automated cropping of prostate magnetic resonance images
Source: J Med Imaging (Bellingham). 2023 Mar 7;10(2):024004. doi: 10.1117/1.JMI.10.2.024004 (PMC9990132; doi:10.1117/1.JMI.10.2.024004)
Supplement: Supplementary file 1 [file JMI_010_024004_SD001.pdf]

Table S1 represents the validation accuracy of the 5 different CNN-based models for different CROPro settings with center cropping. The best performing settings are highlighted in bold.

Cropped Image ($pixels^2$)	Pixel Space (mm^2)	AlexNet	VGG19	ResNet 50	Squeeze Net	DenseNet121
256x256	0.5x0.5	**0.640±0.012**	0.649±0.008	0.598±0.010	0.638±0.005	**0.647±0.005**
256x256	0.4x0.4	0.624±0.006	**0.655±0.009**	0.629±0.004	0.640±0.005	0.632±0.009
256x256	0.3x0.3	0.622±0.012	0.633±0.008	0.619±0.013	0.653±0.003	0.628±0.004
256x256	0.2x0.2	0.631±0.019	0.621±0.017	**0.639±0.006**	0.660±0.010	0.644±0.012
128x128	0.5x0.5	0.603±0.018	0.632±0.010	0.624±0.007	**0.666±0.005**	0.624±0.003
128x128	0.4x0.4	0.588±0.009	0.616±0.020	0.613±0.015	0.640±0.011	0.643±0.011
128x128	0.3x0.3	0.513±0.014	0.559±0.008	0.526±0.013	0.512±0.017	0.546±0.013
128x128	0.2x0.2	0.505±0.013	0.500±0.021	0.524±0.015	0.496±0.023	0.515±0.015
64x64	0.5x0.5	0.538±0.009	0.530±0.017	0.540±0.012	0.526±0.012	0.521±0.014
64x64	0.4x0.4	0.506±0.012	0.492±0.015	0.517±0.008	0.516±0.021	0.498±0.006

Table S2 represents the validation accuracy of the 5 different CNN-based models for different CROPro settings with random cropping. The best performing settings are highlighted in bold.

Cropped Image ($pixels^2$)	Pixel Space (mm^2)	AlexNet	VGG19	ResNet 50	Squeeze Net	DenseNet121
256x256	0.5x0.5	0.641±0.018	0.640±0.008	0.593±0.006	0.637±0.009	0.646±0.007
256x256	0.4x0.4	0.611±0.008	0.625±0.009	0.627±0.005	0.644±0.003	0.630±0.003
256x256	0.3x0.3	**0.642±0.010**	0.639±0.001	0.626±0.008	0.638±0.006	0.628±0.005
256x256	0.2x0.2	0.628±0.008	**0.644±0.018**	**0.649±0.011**	**0.687±0.004**	**0.656±0.006**
128x128	0.5x0.5	0.612±0.009	0.622±0.007	0.614±0.005	0.651±0.005	0.642±0.008
128x128	0.4x0.4	0.613±0.005	0.628±0.009	0.626±0.007	0.659±0.002	0.644±0.006
128x128	0.3x0.3	0.624±0.004	0.595±0.009	0.642±0.005	0.644±0.005	0.630±0.005
128x128	0.2x0.2	0.600±0.012	0.587±0.003	0.619±0.005	0.604±0.004	0.587±0.006
64x64	0.5x0.5	0.610±0.005	0.619±0.008	0.622±0.005	0.616±0.004	0.607±0.008
64x64	0.4x0.4	0.608±0.005	0.596±0.003	0.621±0.004	0.623±0.002	0.604±0.003

Table S3 This table represents the validation accuracy of the 5 different CNN-based models for different CROPro settings with stride cropping. The best performing settings are highlighted in bold.

Cropped Image ($pixels^2$)	Pixel Space (mm^2)	AlexNet	VGG19	ResNet 50	Squeeze Net	DenseNet121
256x256	0.5x0.5	0.626±0.015	0.641±0.006	0.592±0.004	0.640±0.014	0.657±0.010
256x256	0.4x0.4	0.629±0.005	0.648±0.008	0.649±0.007	0.639±0.016	0.644±0.007
256x256	0.3x0.3	0.620±0.007	0.638±0.010	0.626±0.004	0.649±0.011	0.630±0.009
256x256	0.2x0.2	**0.657±0.014**	**0.666±0.006**	**0.670±0.006**	0.691±0.010	**0.658±0.011**
128x128	0.5x0.5	0.622±0.002	0.645±0.007	0.627±0.004	**0.672±0.009**	0.645±0.010
128x128	0.4x0.4	0.617±0.008	0.626±0.019	0.638±0.004	0.661±0.004	0.644±0.003
128x128	0.3x0.3	0.640±0.008	0.603±0.014	0.656±0.008	0.659±0.005	0.640±0.002
128x128	0.2x0.2	0.621±0.010	0.592±0.007	0.635±0.007	0.625±0.004	0.613±0.006
64x64	0.5x0.5	0.615±0.013	0.615±0.008	0.627±0.004	0.611±0.003	0.606±0.003
64x64	0.4x0.4	0.604±0.009	0.589±0.005	0.603±0.008	0.629±0.009	0.612±0.006

Table S4 This table represents the validation accuracy of the 5 different ViT-based models for different CROPro settings with center cropping. The best performing settings are highlighted in bold.

Cropped Image ($pixels^2$)	Pixel Space (mm^2)	ViT-H/14	ViT-L/32	ViT-L/16	ViT-B/32	ViT-B/16
256x256	0.5x0.5	0.620±0.057	0.624±0.056	0.596±0.024	0.640±0.004	**0.663±0.024**
256x256	0.4x0.4	0.640±0.030	0.641±0.033	**0.613±0.030**	0.641±0.016	0.625±0.034
256x256	0.3x0.3	0.624±0.034	0.613±0.025	0.598±0.029	0.624±0.039	0.616±0.031
256x256	0.2x0.2	**0.674±0.016**	0.590±0.063	0.514±0.032	0.589±0.032	0.588±0.052
128x128	0.5x0.5	0.633±0.028	**0.654±0.038**	0.601±0.065	**0.641±0.019**	0.617±0.035
128x128	0.4x0.4	0.653±0.006	0.592±0.048	0.507±0.026	0.577±0.071	0.587±0.048
128x128	0.3x0.3	0.576±0.018	0.534±0.035	0.461±0.047	0.515±0.064	0.519±0.015
128x128	0.2x0.2	0.509±0.011	0.482±0.025	0.460±0.023	0.477±0.025	0.485±0.017
64x64	0.5x0.5	0.545±0.019	0.536±0.015	0.485±0.016	0.483±0.011	0.491±0.011
64x64	0.4x0.4	0.468±0.008	0.480±0.019	0.486±0.025	0.512±0.035	0.534±0.019

Table S5 This table represents the validation accuracy of the 5 different ViT-based models for different CROPro settings with random cropping. The best performing settings are highlighted in bold.

Cropped Image ($pixels^2$)	Pixel Space (mm^2)	ViT-H/14	ViT-L/32	ViT-L/16	ViT-B/32	ViT-B/16
256x256	0.5x0.5	0.667±0.021	0.681±0.045	0.605±0.081	0.651±0.041	0.651±0.029
256x256	0.4x0.4	0.666±0.014	0.646±0.028	0.617±0.020	0.645±0.026	0.650±0.032
256x256	0.3x0.3	0.656±0.015	0.606±0.031	0.616±0.035	0.646±0.010	0.640±0.018
256x256	0.2x0.2	0.728±0.017	0.700±0.027	0.624±0.073	**0.702±0.021**	**0.709±0.029**
128x128	0.5x0.5	0.664±0.046	0.667±0.019	0.634±0.023	0.666±0.015	0.673±0.012
128x128	0.4x0.4	0.714±0.013	**0.709±0.020**	0.620±0.034	0.697±0.013	0.707±0.053
128x128	0.3x0.3	0.737±0.017	0.696±0.032	**0.649±0.019**	0.686±0.032	0.707±0.037
128x128	0.2x0.2	**0.741±0.008**	0.684±0.048	0.640±0.013	0.669±0.011	0.670±0.034
64x64	0.5x0.5	0.732±0.015	0.707±0.033	0.635±0.012	0.698±0.029	0.671±0.041
64x64	0.4x0.4	0.721±0.013	0.654±0.020	0.626±0.016	0.667±0.012	0.665±0.026

Table S6 This table represents the validation accuracy of the 5 different ViT-based models for different CROPro settings with stride cropping. The best performing settings are highlighted in bold.

Cropped Image ($pixels^2$)	Pixel Space (mm^2)	ViT-H/14	ViT-L/32	ViT-L/16	ViT-B/32	ViT-B/16
256x256	0.5x0.5	0.661±0.025	0.654±0.030	0.542±0.032	0.667±0.011	0.686±0.028
256x256	0.4x0.4	0.661±0.006	0.612±0.033	0.574±0.058	0.609±0.063	0.638±0.032
256x256	0.3x0.3	0.650±0.023	0.620±0.039	0.562±0.061	0.643±0.016	0.616±0.031
256x256	0.2x0.2	0.714±0.026	0.690±0.016	0.663±0.030	0.690±0.015	0.695±0.040
128x128	0.5x0.5	0.653±0.035	0.646±0.037	0.578±0.070	0.630±0.031	0.606±0.055
128x128	0.4x0.4	0.715±0.011	0.649±0.040	0.593±0.055	0.653±0.025	0.672±0.050
128x128	0.3x0.3	0.694±0.029	0.709±0.015	**0.667±0.008**	**0.714±0.022**	0.689±0.014
128x128	0.2x0.2	**0.733±0.004**	**0.719±0.031**	0.656±0.004	0.687±0.033	**0.729±0.020**
64x64	0.5x0.5	0.713±0.012	0.678±0.052	0.634±0.029	0.648±0.024	0.659±0.042
64x64	0.4x0.4	0.702±0.005	0.662±0.041	0.632±0.048	0.655±0.048	0.642±0.029

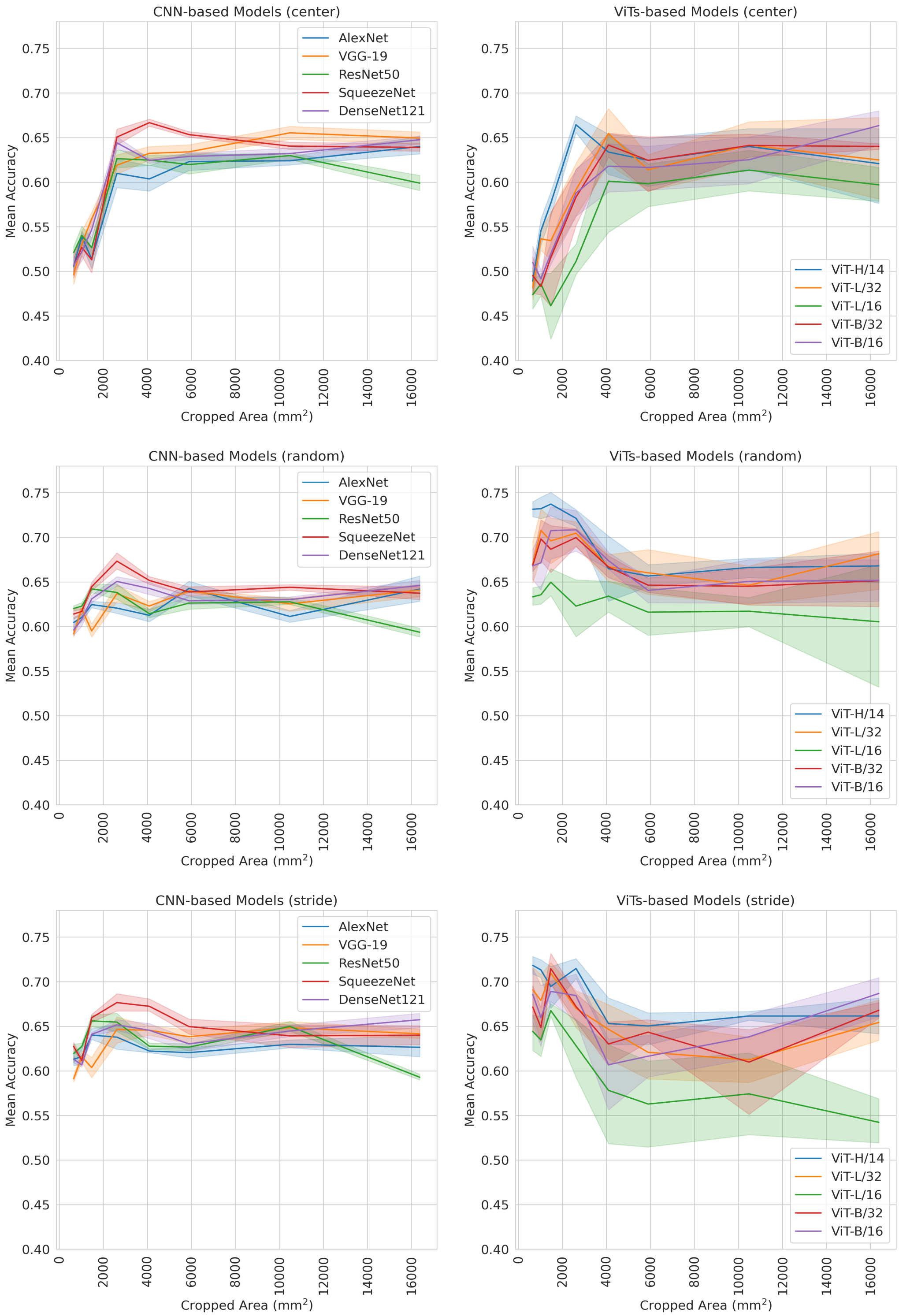

Fig S1 shows the performance of each sampling technique (center, random, stride) for all CNN and ViT-based models, as a function of the area of the cropped images. The solid lines represents the mean accuracy and the shaded areas the 95% confidence intervals.
